# An analysis of digging anchor machine stability and track wear under digging conditions

**DOI:** 10.1038/s41598-022-22738-4

**Published:** 2022-10-22

**Authors:** Xinwei Yang, Dongxuan Wu, Xianfeng Zou, Hongyue Chen, Shuai Zhang

**Affiliations:** 1 School of Mechanical Engineering, Liaoning Technical University, Fuxin, 123000 Liaoning China; 2 Research Institute of Technology and Equipment for the Exploitation and Utilization of Mineral Resources, Fuxin, 123000 Liaoning China

**Keywords:** Mechanical engineering, Statistical physics, thermodynamics and nonlinear dynamics

## Abstract

To analyse the stability of a digging anchor machine under digging conditions, the dynamics model of the anchor machine and the interaction mechanics model between its tracks and the roadway floor are constructed with a Sandvik MB670-1 digging anchor machine in the context of the Zhang Jiamao 5^–2^ coal seam of the Shaanxi Coal and Chemical Industry Group. The discrete element method (DEM) and multibody dynamics (MBD) two-way coupling algorithm is used to simulate the cutting process of a full coal seam and coal rock containing gangue by using a digging anchor machine in a roadway. The changes in the cutting depth of the digging anchor machine drum, the sliding distance of the track and the stress‒strain pattern of the roadway floor are obtained. Finally, through shear slip testing, the stress distribution and deformation pattern of the roadway floor under different grouser parameters of the digging anchor machine track shoe, as well as the wear characteristics of the track shoe, are obtained. The results of this study can provide a theoretical basis for the control and reliability of the digging anchor machine and the life fatigue prediction of its tracks.

## Introduction

The phenomenon of “focus on coal mining, light digging” has existed for a long time, and the imbalance between digging speed and mining speed has become increasingly prominent; this imbalance is a significant factor restricting the safe and efficient production of coal mines. To solve the problem of the low degree of equipment intelligence and the low digging efficiency of comprehensive excavation faces in current roadways, many coal mining enterprises have begun to introduce comprehensive excavation equipment, such as digging anchor machines. Therefore, the stability of a digging anchor machine under digging conditions and its impact on coal mine roadway floors have become the primary reference factors for introducing these machines. Chen Hongyue et al.^1^ analysed the motion characteristics and stability of a digging anchor machine under different geological conditions with the Zhang Jiamao 5^–2^ coal seam of the Shaanxi Coal and Chemical Industry Group and Sandvik MB670-1 as the research object. To more comprehensively analyse the stability of a digging anchor machine in a coal mine roadway and the bearing characteristics of the roadway floor, additional research on the stability of the machine and the bearing characteristics of the floor under digging conditions is needed to provide a basis for ensuring safe and efficient production in coal mines and improving the stability control of the digging anchor machine.

Bekker's research is the most representative with regard to studies of tracked vehicle terramechanics, and the stress‒strain equations for soil shear and pressure-sinkage properties have been successively derived and widely applied^2^. With the development of computer technology, researchers have begun to widely use computer-aided techniques to study vehicle terramechanics problems, and the deformation and stress distribution of soil under tractor wheels were obtained for the first time^3,4^. The flexible track model (NTVPM model) for high-speed tracked vehicles and the rigid track model (RTVPM model) for low-speed tracked vehicles were proposed to characterize the ground pressure distribution under different motion characteristics of tracked vehicles^5–9^. After entering the twenty-first century, research on vehicle terramechanics has undergone rapid development, and track-ground interaction models^10^, tracked vehicle soft ground passing performance models^11^, and methods for predicting the driving resistance of tracked vehicles^12^ have been proposed. The travel characteristics of tracked vehicles of different structural dimensions under different operating conditions have been obtained^13^. Meanwhile, the dynamics of the lunar rover during driving processes was simulated^14^. In recent years, several experts and scholars have conducted an increasing number of research studies regarding the process of driving tracked vehicles on soft surfaces. Madsen et al.^15^ conducted a physics-based simulation analysis of vehicle/terrain interactions using an off-road vehicle model. Azimi et al.^16^ performed the interactive simulation and analysis of planetary wheels. He Jian et al.^17^ comprehensively considered the shear stress, loading and unloading processes and loading and unloading rate factors to verify the accuracy of a new bearing model to simulate the vibration characteristics of a tracked vehicle. By measuring vertical and horizontal stresses 35 cm below a type of self-propelled half-track harvester, Ding Zhao et al.^18^ obtained normal and tangential force distribution patterns of a tracked vehicle with respect to the ground, which provided a theoretical basis for optimizing the tracked travel mechanism. Zhou et al.^19,20^ investigated the dynamic characteristics of tracked vehicle loading wheels using a multibody dynamics simulation method. Zhou et al.^21–23^ proposed a sandstone modelling method based on three-dimensional computer scanning and colour enhancement technique, and used a coupling multiple point statistic-marching cube three-dimensional reconstruction algorithm to analyze the effect of microscopic heterogeneity of sandstone on the damage properties.

A review of the above studies and findings reveals the dynamic characteristics of tracked vehicles and track-ground interactions under static or quasi-static conditions on nondeformable ground. Due to the large size of the digging anchor machine and the lengthy walking track, it is necessary to consider the overall flexible deformation of the track when studying the stability of the digging anchor machine and track-roadway floor interactions under digging conditions. Furthermore, due to the harsh working environment in the underground roadway of a coal mine, the structural characteristics of the roadway floor are different from those of the surface, so traditional research methods cannot ensure result accuracy. In this paper, based on the background of the Zhang Jiamao 5^–2^ coal seam of the Shaanxi Coal and Chemical Industry Group, a Sandvik MB670-1 digging anchor machine is used as the research object, and a two-way coupling technique of the discrete element method (DEM) and multibody dynamics (MBD) method is adopted to simulate the cutting process of a full coal seam and coal rock containing gangue in the roadway by the digging anchor machine. The change patterns of the digging anchor machine cutting depth, track sliding distance and wear degree, and the stress‒strain of the roadway floor are analysed. The results of this study can provide a theoretical basis for the control and reliability of a digging anchor machine and the structural design and life fatigue prediction of its tracks.

## Model building

### Digging anchor machine model building

**Figure 1:**
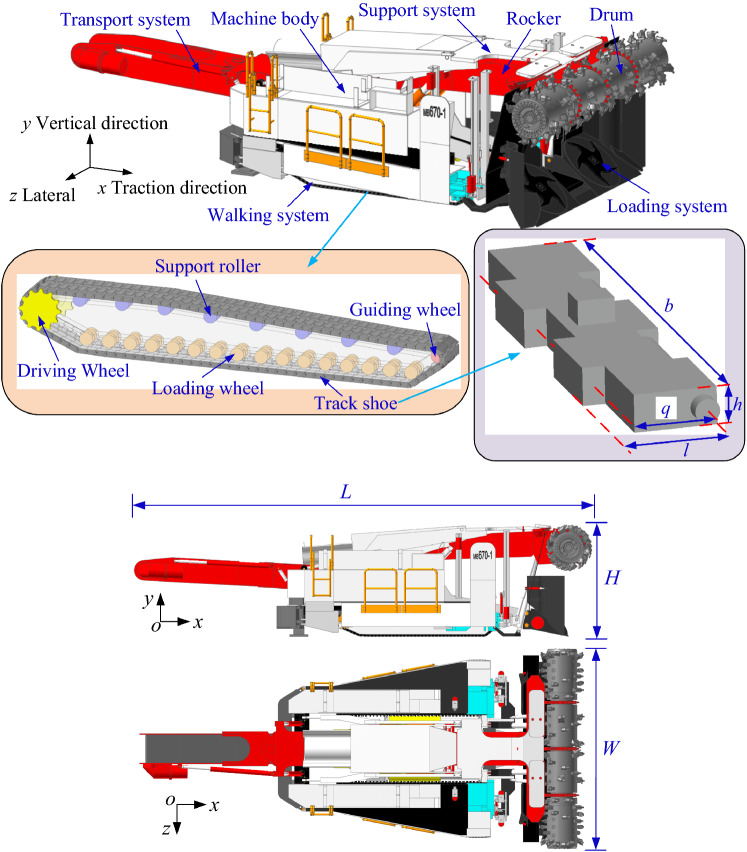
Digging anchor machine model.

An MB670 digging anchor machine is selected as the research object, and the digging anchor machine is divided into seven parts: a drum, a rocker, a support system, a loading system, a transportation system, a walking system and the machine body. When building a geometric model, the walking chassis is simplified, and the motor, reducer and frame are simplified into a rigid body. Finally, the chassis is simplified into two parts: a connecting frame and a tracking subsystem. The whole structure is shown in Fig. 1. The main technical parameters of the digging anchor machine are shown in Table 1, and the main parameters of the track shoe are shown in Table 2.

**Table 1:** Digging anchor machine parameters.

Parameters	Values
Overall weight/t	105
Dimension (L × W × H)/mm	11,250 × 4900 × 2650
Drum diameter/mm	1150
Drum width/mm	5200
length of track on ground/mm	3100
Ground pressure/kPa	280

**Table 2:** Track shoe main parameters.

Pitch q/mm	Length l/mm	Width b/mm	Thickness h/mm
89.04	108	605	46.12

### Floor particle bed model building

Based on the density, water content, uniaxial tension, uniaxial compression, and shear tests conducted on physical and mechanical parameters of the Zhang Jiamao 5^–2^ coal seam and roof strata according to the literature^24^, the natural coal sample and natural mudstone in the experiment are selected as simulation materials, and corresponding parameters are obtained as shown in Table 3.

**Table 3:** Physical and mechanical parameters of the materials.

Materials	Natural coal sample	Natural mudstone
Density ρ/(kg·m^−3^)	1300	2350
Poisson's ratio μ	0.31	0.23
Shear modulus G/Pa	1.20 × 10^9^	6.80 × 10^9^
Elastic modulus E/Pa	3.22 × 10^9^	1.67 × 10^10^
Compressive strength σ_1_/Pa	2.39 × 10^7^	4.97 × 10^7^
Tensile strength σ_2_/Pa	6.90 × 10^5^	1.17 × 10^6^
Internal cohesion c/Pa	5.79 × 10^6^	7.95 × 10^6^
Internal friction angle φ/(°)	33.67	23.27

The floor particle bed model is built with the above parameters. It is also necessary to set contact parameters between materials, including the restitution coefficient, static friction coefficient and dynamic friction coefficient. According to the literature^25,26^, the contact parameters between coal rock are listed in Table 4.

**Table 4:** Contact parameters between materials.

Contact parameters	Restitution coefficient	Static friction coefficient	Dynamic friction coefficient
Natural coal sample—Natural coal sample	0.5	0.6	0.05
Natural mudstone—Natural mudstone	0.6	0.8	0.1
Natural coal sample—Natural mudstone	0.55	0.7	0.08

#### Particle contact model

**Figure 2:**
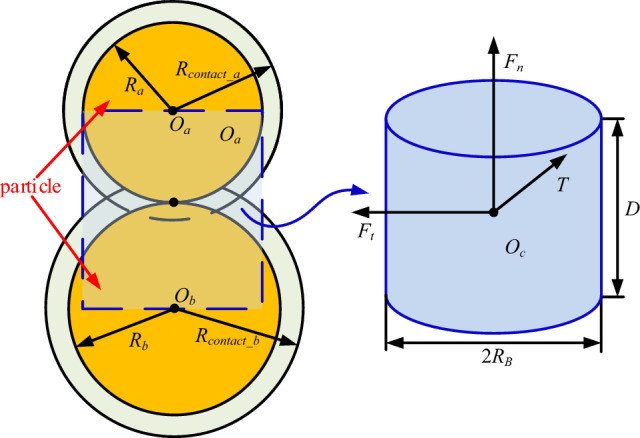
Particle adhesive model.

Considering the practical situation in which the EDEM (discrete element method) provides a variety of applicable interparticle contact models, the bonding contact model is used to simulate the adhesion and fragmentation process of coal rock particles^27^. When adhesion occurs between two standard spherical particles *a* and *b* with particle radii *R*_*a*_ and *R*_*b*_, respectively; the particle adhesive model is shown in Fig. 2. The dashed area in the figure shows the cylindrical beam of the adhesive structure. The cylindrical beam is a virtual area with *O*_*c*_ as the centre of the cylinder, the radius of adhesion *R*_*B*_ as the radius of the cylinder, and the length of adhesive bond *D* as the height of the cylinder. In this region, forces and moments can be transferred between particles in contact with each other, and certain tangential and normal motions can be withstood. When adhesive bonds are generated, normal forces, tangential forces, normal moments, and tangential moments on the coal rock particles are initialized. After that, the magnitudes of the force and moment are updated in real time by computer simulation as the time step increases, and the adhesive force and adhesive moment in the simulation software are updated by Eq. (1):

1 $$  \left\{ \begin{gathered}   \delta F_{n}  =  - v_{n} S_{n} A\delta _{t}  \hfill \\   \delta F_{t}  =  - v_{t} S_{t} A\delta _{t}  \hfill \\   \delta F_{n}  =  - \omega _{n} S_{n} J\delta _{t}  \hfill \\   \delta T_{t}  =  - \omega _{t} S_{n} \frac{J}{2}\delta _{t}  \hfill \\   A = \pi R_{B}^{2}  \hfill \\   J = \frac{1}{2}\pi R_{B}^{4}  \hfill \\  \end{gathered}  \right.  $$

where *δF*_*n*_ and *δF*_*t*_ are normal and tangential forces of the corresponding computational step of the adhesive bond, respectively; *δT*_*n*_ and *δT*_*t*_ are normal and tangential moments of the corresponding computational step of the adhesive bond, respectively; *δ*_*t*_ is the simulation step size; *S*_*n*_ and *S*_*t*_ are the normal and tangential stiffnesses per unit area of the adhesive bond, respectively; *v*_*n*_ and *v*_*t*_ are the normal and tangential velocities of the particle, respectively; *ω*_*n*_ and *ω*_*t*_ are the normal and tangential rotational angular velocity of the particle, respectively; *A* is the cross-sectional area of the adhesive bond region; *J* is the polar moment of inertia of the adhesive bond; and *R*_*B*_ is the radius of the adhesive bond region.

When the interparticle stress exceeds the adhesive strength, the adhesive bond is destroyed; at this time, the ultimate normal and tangential stresses of the interparticle adhesive bond can be described by the following:

2 $$ \left\{ \begin{gathered} \sigma_{\max } < - \frac{{F_{n} }}{A} + \frac{{2T_{t} }}{J}R_{B} \hfill \\ \tau_{\max } < - \frac{{F_{t} }}{A} + \frac{{T_{n} }}{J}R_{B} \hfill \\ \end{gathered} \right. $$

where *σ*_max_ is the maximum normal stress of the adhesive bond and *τ* is the maximum tangential stress of the adhesive bond.

By setting the particle contact radius *R*_*contact*_, the region of the adhesive bond is determined. To avoid particle gaps that prevent the generation of proper adhesive bonds, the particle contact radius should always be larger than its physical radius. Typically, the contact radius is taken to be 1.1 to 1.3 times the physical radius. If the contact radius is less than this range, then the brittleness of the particle geometry will be increased, and conversely, if the contact radius is taken to be greater than this range, then the adhesive bonds between unrelated particles will be created. Therefore, the simulation sets the ratio of the interparticle contact radius to the physical radius to 1:1.

The force–displacement behaviour of each adhesive bond part is mainly described by five parameters, namely, the normal and tangential stiffnesses per unit area, i.e., Sn and St; the critical stresses, i.e., σ and τ; and the radius of the adhesive bond area *R*_*B*_. These parameters determine the adhesive effect of the parallel adhesive model. The normal and tangential stiffnesses and normal and tangential strength of the adhesive bond can be found by referring to the prediction formula proposed by Potyondy et al.^28^ that can be fitted to the rock mechanics parameters.

##### Determination of the per unit area stiffness

The normal stiffness per unit area represents the tensile or compressive stiffness along the principal axis of adhesion, and the tangential stiffness per unit area represents the shear stiffness in the plane orthogonal to the principal axis of bond. When the ratio of tangential stiffness to normal stiffness of the adhesive bond is defined as the stiffness conversion Factor *k*, the solution formula is as follows^28^:

3 $$ \left\{ \begin{gathered} S_{n} = \frac{{E^{*} }}{D} \hfill \\ S_{t} = kS_{n} \hfill \\ \end{gathered} \right. $$

where E^*^ is the equivalent elastic modulus and *k* is the stiffness conversion factor, which is taken as 0.4.

4 $$ \frac{1}{{E^{*} }} = \frac{{1 - \mu_{a}^{2} }}{{E_{a} }} + \frac{{1 - \mu_{b}^{2} }}{{E_{b} }} $$

where *E*_*a*_ and *E*_*b*_ are the elastic moduli of coal rock and gangue, respectively, and $\mu_{a}$ and $\mu_{b}$ are the Poisson's ratios of coal rock and gangue, respectively.

##### Determination of the critical stress

According to the calculation method of the adhesion coefficient, the critical normal stress of the adhesive bond is expressed as the new ultimate tensile strength of coal rock under adhesive conditions, and the critical tangential stress of the adhesive bond is expressed as the new ultimate shear strength of coal rock under adhesive conditions. By introducing the cohesive force and internal friction angle of coal rock, the strain strength applicable to adhesive solid material is obtained as:

5 $$ \left\{ \begin{gathered} \hat{\sigma }_{1} = \sigma_{1} + \sigma_{0} \hfill \\ \hat{\sigma }_{2} = \sigma_{2} + \sigma_{0} \hfill \\ \hat{\tau }_{1} = \tau + \sigma_{0} \hfill \\ \end{gathered} \right. $$

where $\hat{\sigma }_{1}$ is the new ultimate compressive strength under adhesive conditions; $\hat{\sigma }_{2}$ is the new ultimate tensile strength under adhesive conditions; $\hat{\tau }_{1}$ is the new ultimate shear strength under adhesive conditions; and $\sigma_{0}$ is the additional three-way strength under adhesive conditions.

6 $$ \left\{ \begin{gathered} \tau = c + \sigma_{1} \tan \phi \hfill \\ \sigma_{0} = c\cot \phi \hfill \\ \end{gathered} \right. $$

where *c* is the cohesive force of the coal rock and *φ* is the internal friction angle of the coal rock.

##### Determination of the adhesive region radius

The adhesive region radius is the radius of the cylindrical bond between particles. A larger volume in this region indicates a stronger bond, and the adhesive region radius can be obtained from the following equation:

7 $$ R_{B} = \lambda \min \left( {R_{a} ,R_{b} } \right) $$

where *λ* is the adhesive bond radius multiplier, taken as 0.5.

Based on Eqs. (3) to (7) and known coal rock parameters, the calculated coal rock particle adhesion parameters are shown in Table 5.

**Table 5:** Particle adhesion parameters.

Particle adhesion parameters	Coal- Coal	Gangue- Gangue	Coal- Gangue
*S*_*n*_/(N/m^3^)	4.5 × 10^10^	2.9 × 10^11^	8.6 × 10^10^
*S*_*t*_/(N/m^3^)	1.8 × 10^10^	1.5 × 10^11^	4.3 × 10^10^
*σ*/Pa	3.2 × 10^6^	2.0 × 10^7^	1.5 × 10^7^
*τ*/Pa	3.0 × 10^6^	4.8 × 10^7^	3.9 × 10^7^

**Figure 3:**
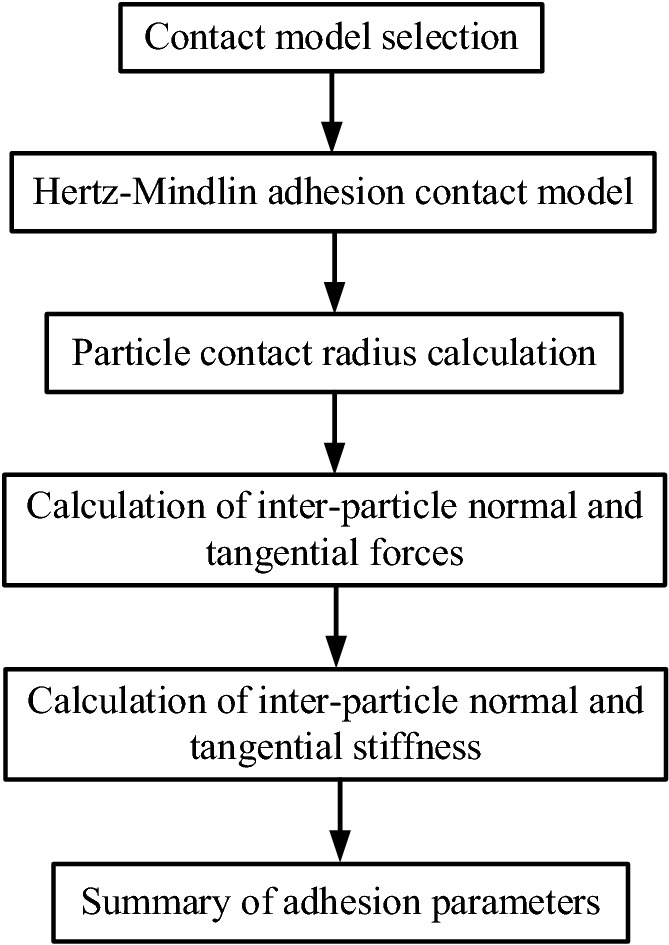
Flow chart of the interparticle contact model calculation.

The flow chart of the interparticle contact model calculation is shown in Fig. 3.

**Figure 4:**
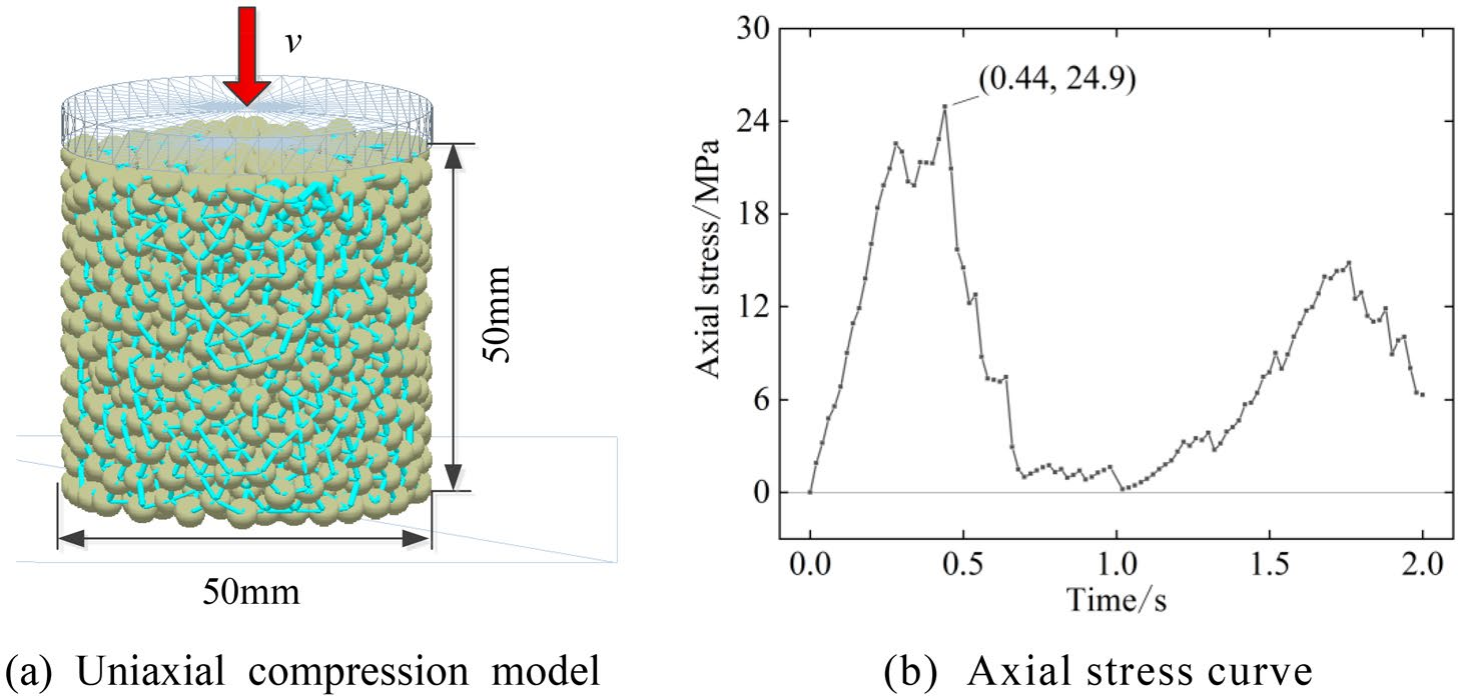
Uniaxial compression test model and results.

To verify the validity of the model, a uniaxial compression simulation test is established, as shown in Fig. 4, where the coal rock specimen is a cylinder with a diameter of 50 mm and a height of 50 mm. The specimen model is shown in Fig. 4a. The axial force is applied to the cylinder until the specimen is broken, and the axial stress curve is obtained, as shown in Fig. 4b. The maximum axial stress is 24.9 MPa, which is within a 5% error of the compressive strength in Table 2. Therefore, the discrete element model of the floor can simulate the actual floor properties.

#### Track-floor wear model

The Archard wear model^29^ is a wear model for particle-geometry contact that simulates the wear at various locations during track shoe movement. The wear depth of the geometric surface can be obtained using the EDEM with the help of this model, and the wear model equation is as follows:

8 $$ \Delta h = \frac{K \cdot p}{H}\Delta s $$

where Δ*h* is the material wear depth; *K* is the dimensionless wear constant, where the wear constant for steel is set at 1 × 10^–12^ depending on the type of track material; *p* is the normal stress on the track shoe surface; *H* is the material surface hardness; and Δ*s* is the particle slip distance.

The accumulation of contact energy in the normal direction is known as normal impact wear and is calculated by the following equation:

9 $$ E_{n} = \sum {\left| {F_{ni} v_{n} \delta_{t} } \right|} $$

where *F*_*ni*_ is the normal force of the track.

The accumulation of contact energy in the tangential direction is known as tangential impact wear and is calculated by the following equation:

10 $$ E_{t} = \sum {\left| {F_{ti} v_{t} \delta_{t} } \right|} $$

where *F*_*ti*_ is the tangential force of the track.

## Analysis of track dynamic characteristics under cutting conditions

**Figure 5:**
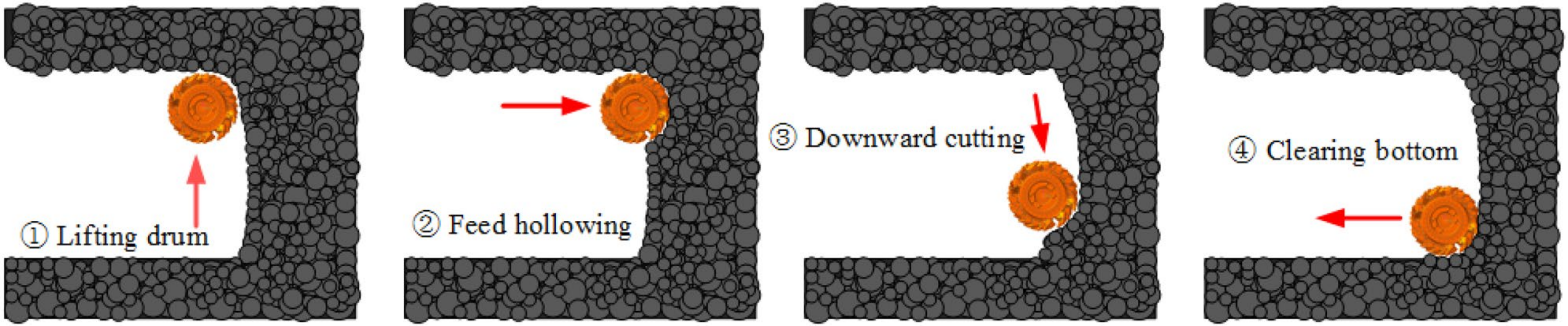
Cutting operation flow.

As shown in Fig. 5, the single feed operation flow of the comprehensive excavation face related to the cutting process is divided into four main stages: ① start the drum and lift the cutting arm to the top of the roadway, ② the pushing cylinder drives the cutting head to realize hollowing action, ③ the cutting arm swings down and cuts in the order of top to bottom, and ④ the pushing cylinder drives the cutting head to clear the bottom. Since feed hollowing and downwards cutting are the main components of the cutting operation and occupy most of the time in digging conditions, the following cutting simulation analysis is performed only for the second and third stages.

### Cutting coupling model

#### Parameters of the cutting system

**Figure 6:**
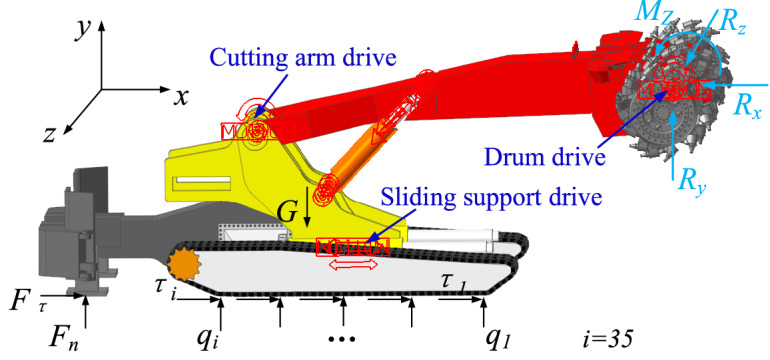
Cutting dynamics model.

The drive imposed on the digging anchor machine dynamics model is shown in Fig. 6. No power is output from the left and right tracks of the digging anchor machine, and together with rear support, the stability of digging anchor machine is maintained during cutting. The drum rotates clockwise at the maximum angular speed to cut the coal rock, and the drum position is controlled by the end of the cutting arm. The pushing cylinder drives the support frame, cutting arm and drum to move forward horizontally to realize hollowing action. The cutting arm swings down around the shaft and drives the drum to achieve downwards cutting. If a hydraulic cylinder is used to drive cutting arm movement, then the driving function will be very complicated. Therefore, the power is set at the hinge between the cutting arm and sliding support, and the drum rotates clockwise around the rotation axis.

Using the distance *d*_2_ from the drum axis to the cutting arm rotation axis in the model as the cutting rotation radius, the angular velocity *ω*_2_ of the downwards swing can be found according to Eq. (11):

11 $$ \omega_{2} = \frac{{v_{2} }}{{d_{2} }} $$

where *v*_2_ is the maximum downwards speed.

The angular velocity of drum rotation *ω*_3_ is found by Eq. (12):

12 $$ \omega_{3} = 2\pi f $$

where *f* is the drum rotation speed.

The cutting parameters are shown in Table 6, and the driving parameters can be solved jointly by data in the table, where the hollowing distance in the simulation is taken as one-half of the maximum hollowing distance and the drive function type is displacement when hollowing to simulate the start and stop of pushing cylinder. To realize the virtual prototype to hollow first and then cut coal, the cutting drum angular velocity function is set as *step*(*time*, 0, 0, 0.5, − 2.61), and the pushing displacement function is set as *step*(*time*, 0, 0, 10, 500). After hollowing is finished, downwards cutting starts, and the downwards angular velocity function of the cutting arm is *step* (*time*, 10, 0, 10.5, − 0.012).

**Table 6:** Cutting parameters table.

Cutting section shape	Cutting width	Cutting height	Drum diameter	Drum rotation speed
Rectangle	5.2 m	2.8–3.6 m	1.15 m	25 rpm
Maximum hollowing and download speed	Cutting arm rotation radius	Angular speed of the cutting arm swing down	Angular velocity of drum rotation	Maximum hollowing distance
0.05 m/s	4.8 m	0.012 rad/s	2.6 1 rad/s	1 m

#### Coupling model

**Figure 7:**
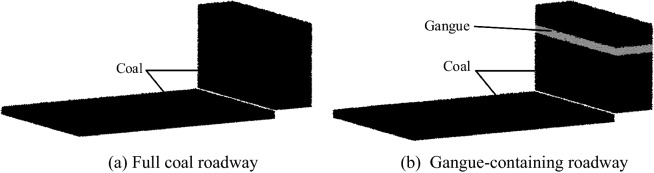
Roadway model.

Based on geological survey results from the literature^24^ in Zhang Jiamao coal mining, the roadway particle bed model is shown in Fig. 7. The roadway model consists of two parts: a floor and a body filled with coal particles. The floor is 8 m long and 5.5 m wide, and the body filled with coal particles is 0.3 m thick. The particles have a 25 mm radius and a 28 mm contact radius. The coal wall is 5.5 m wide and 3 m high, and its body is filled with a 1.5 m thick coal rock layer. The coal wall section is filled with natural coal, with 25 mm coal particles. There is 0.3 m gangue layer inside coal wall at a height of 2.5 m. The gangue particles have a 20 mm radius and a 22 mm contact radius. The remaining space is filled with coal rock.

**Figure 8:**
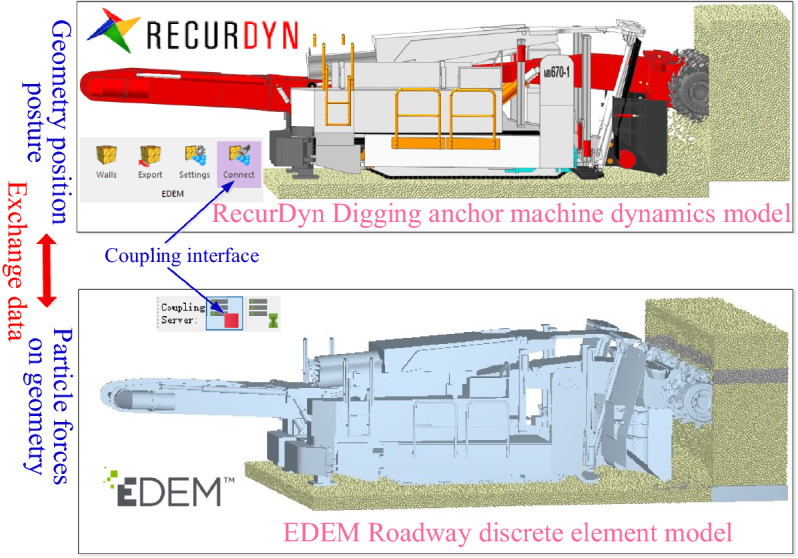
Cutting coupling model.

The simulation time is set to 20 s, the simulation time step is taken as 25% in EDEM (discrete element method), and the save interval is set to 0.05 s in RecurDyn. As shown in Fig. 8, the discrete element model of the roadway can be associated with the virtual prototype model of digging anchor machine by means of the coupling interface between EDEM (discrete element method) and RecurDyn (recursive dynamic). During the simulation process, coal rock state information and digging anchor machine movement information are transmitted in real time, ensuring that the forces of particles on the geometry and geometry position can restore a realistic cutting scene to the greatest extent and narrow the gap between simulation data and real cutting data.

### Analysis of the simulation results

#### Cutting drum load

**Figure 9:**
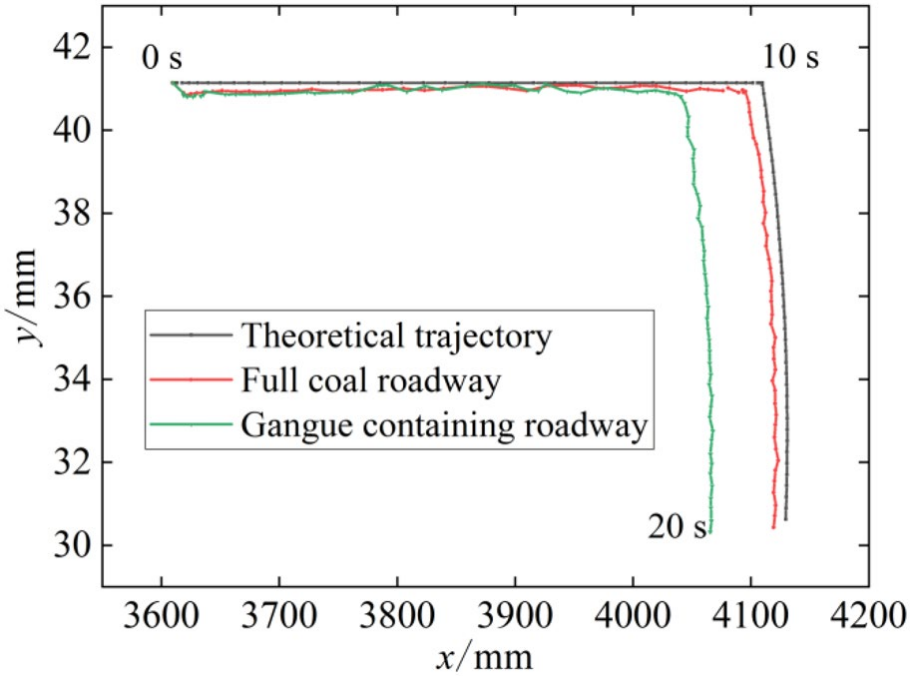
Drum centroid trajectory.

To analyse the influence of a load on drum cutting, the theoretical trajectory of the drum is simulated by using RecurDyn software under the no-load condition of the digging anchor machine. This result is compared with the cutting trajectory of the drum under a full coal seam and a coal seam containing gangue. Figure 9 shows the drum centroid trajectory, in which the theoretical hollowing depth is 500 mm and the theoretical downwards distance is 564 mm. As seen from the figure, the actual height of the centroid during hollowing is always lower than the theoretical value. This is because the whole centroid of the digging anchor machine is forward, and the body is inclined forward after the simulation starts, resulting in a lower drum position. As the hollowing speed increases first and then decreases, the hollowing volume per unit time of drum increases first and then decreases, the cutting resistance in the vertical direction increases first and then decreases, and the direction of resistance is opposite to the direction of rotation. Therefore, the drum height in the cutting stage increases slowly and then decreases slowly under the action of the cutting resistance. Compared with the theoretical value, the error of cutting downwards in both types of roadways is no more than 3 mm, while the gap of the hollowing depth is large. Additionally, the hollowing depth in the full coal roadway is 484 mm, and the hollowing depth in the gangue-containing roadway is 431 mm, which is nearly 10% less than the full coal roadway. Therefore, the existence of the gangue layer increases the horizontal load of the digging anchor machine and reduces the cutting efficiency.

**Figure 10:**
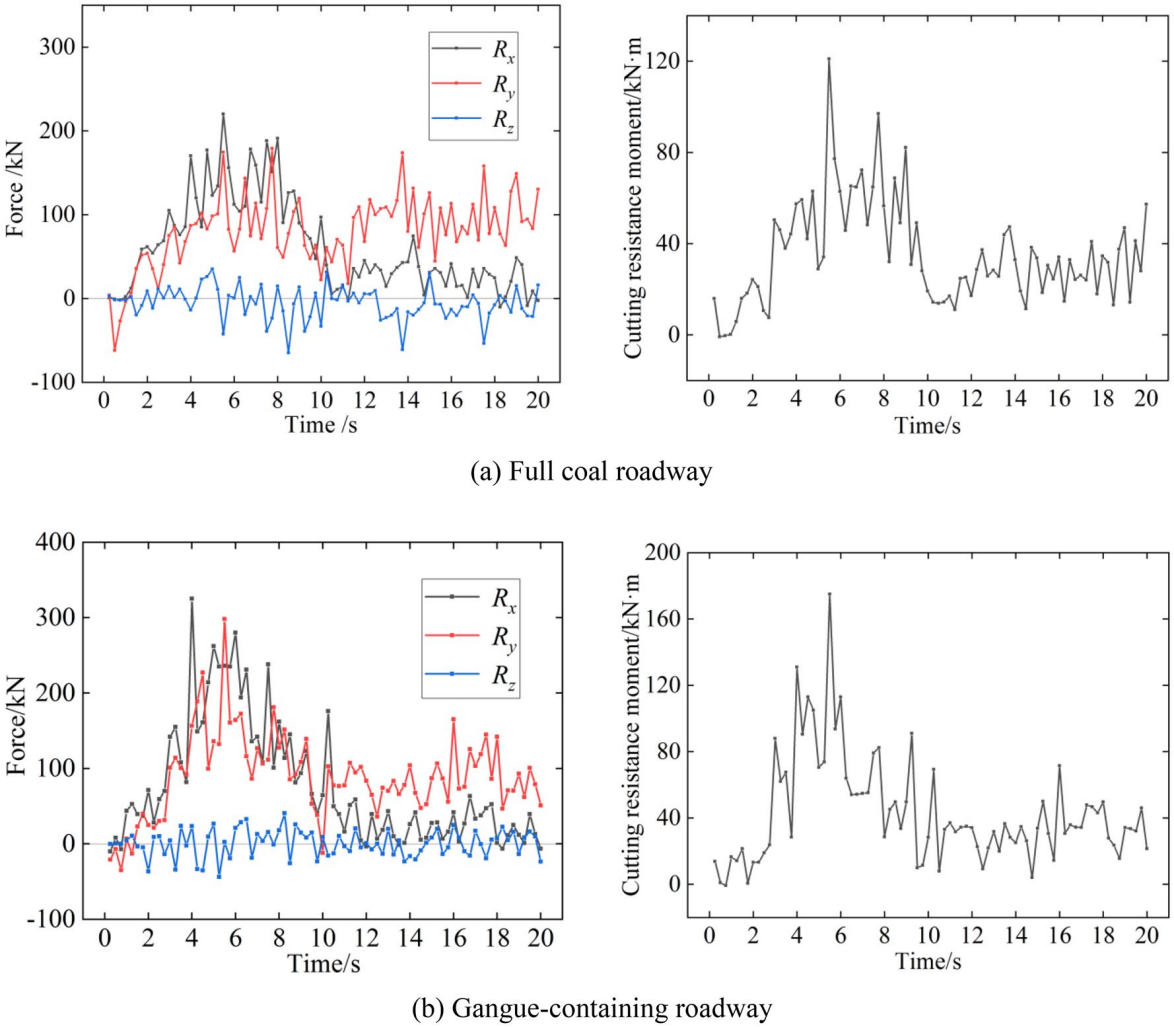
Drum cutting load.

The action of the coal wall on the cutting drum mainly includes the combined forces *R*_*x*_, *R*_*y*_ and *R*_*z*_ of the forces on the *x*, *y* and *z* axes of the cutting teeth and the resistance moment *M*_*z*_ around the drum axis. According to Fig. 10, when the digging anchor machine drum cuts coal rock, the external force on the drum is constantly fluctuating due to the uneven distribution of the cutting teeth and the random distribution characteristics of the coal seam particle filling process. In the feed hollowing stage, *R*_*x*_ > *R*_*y*_ > *R*_*z*_, and the downwards cutting stage, *R*_*y*_ > *R*_*x*_ > *R*_*z*_, and the peaks of the three-way force and resistance moment are shown in Table 7. The drum hollowing depth is less than the theoretical depth, and the cutting depth when the digging anchor machine cuts down the coal wall containing gangue is less than that of the full coal wall, so the cutting force and moment after 10 s are less than those of the full coal roadway.

**Table 7:** Peak drum load.

Peak value	Full coal roadway	Gangue-containing roadway
*R*_*x*_/kN	220	325
*R*_*y*_/kN	197	298
*R*_*z*_/kN	64	43
*M*_*z*_/(kN·m)	121	175

#### Body stability

**Figure 11:**
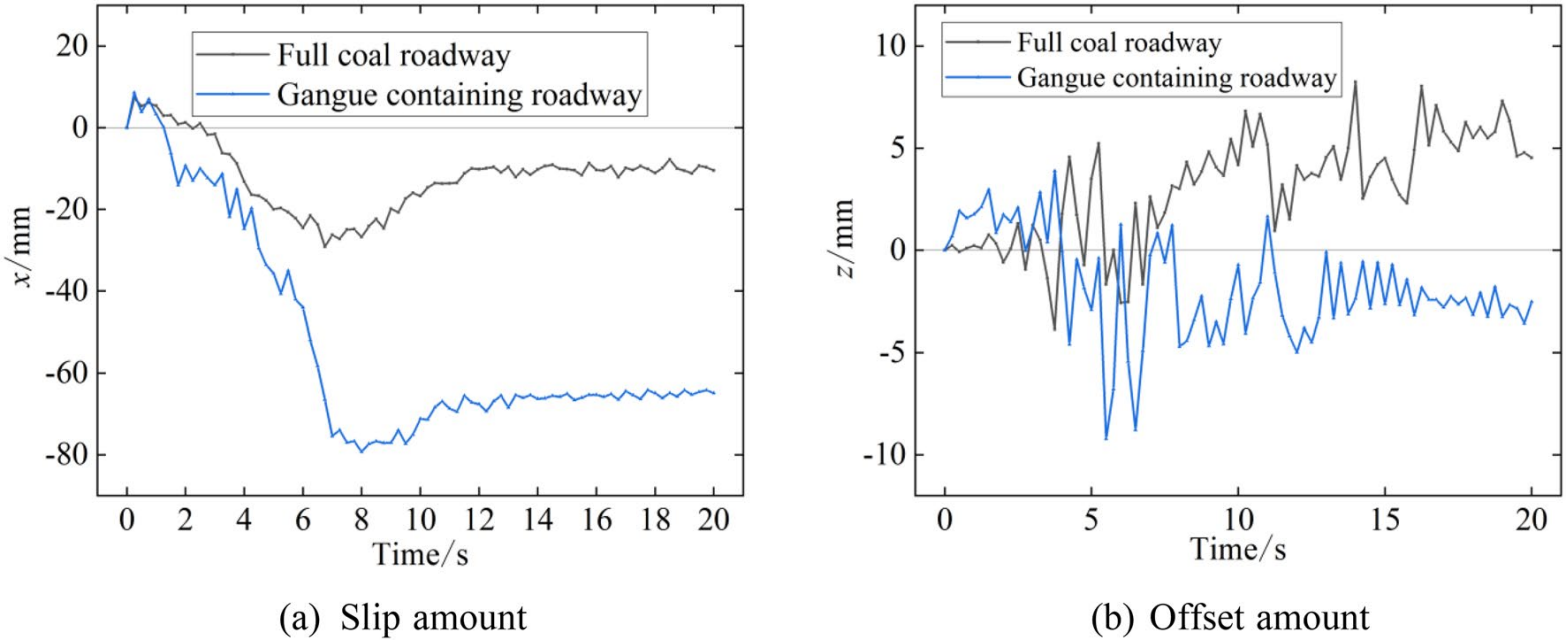
Body displacement.

The drum not only affects its own stability when it interacts with coal rock but also influences the body through the cutting arm and moving support and then from the main frame to the grounding mechanism (rear support and track). Figure 11 shows the displacement of the digging anchor machine in the traction direction. The displacement curve of the centroid in the two types of roadways has a consistent trend of change; that is, the digging anchor machine first moves away from the coal wall and then moves closer to the coal wall while realizing hollowing, and the distance between the centroid and coal wall remains basically constant when it cuts downwards. Movement away from the coal wall is caused by drum cutting resistance, and the positive displacement of the digging anchor machine in the first 2 s and the positive movement in the second half of hollowing are due to a reduction in the pitch angle of the body. At the end of the simulation, the digging anchor machine moved backwards 10 mm in the full coal roadway, and this value reached 65 mm in the gangue-containing roadway, which is 6.5 times higher than that in the full coal roadway.

The larger the lateral displacement of the digging anchor machine in the digging process is, the more floor is subjected to lateral shear. This will cause impact loads when fed back to the drum and will also affect the smoothness on both sides of the roadway. According to the lateral displacement of the centroid for the digging anchor machine during the simulation given in Fig. 11, the lateral displacement of the digging anchor machine in the two types of roadways is between ± 10 mm, which shows that the digging anchor machine is little affected by the lateral load during cutting.

#### Floor deformation by force

**Figure 12:**
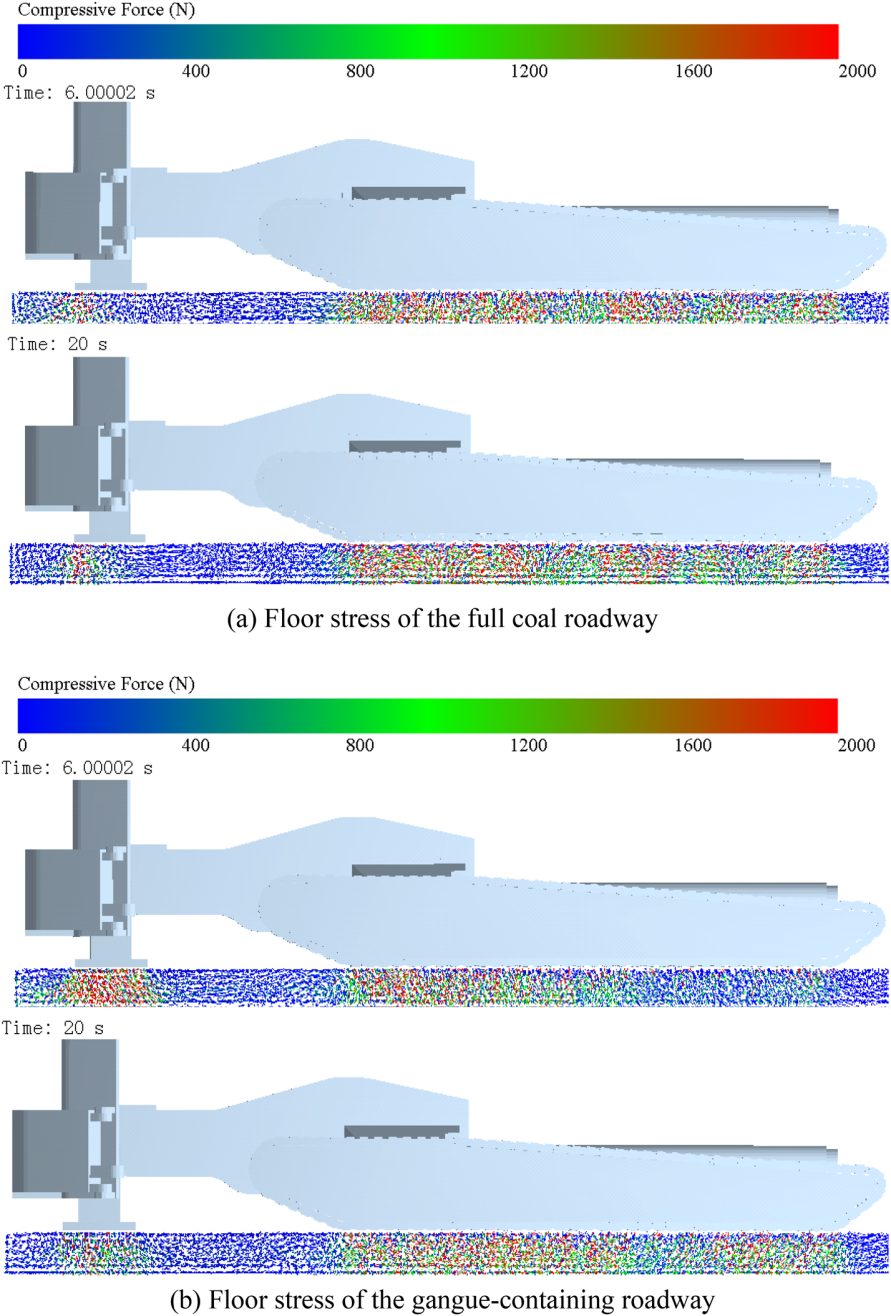
Force distribution of the floor.

**Figure 13:**
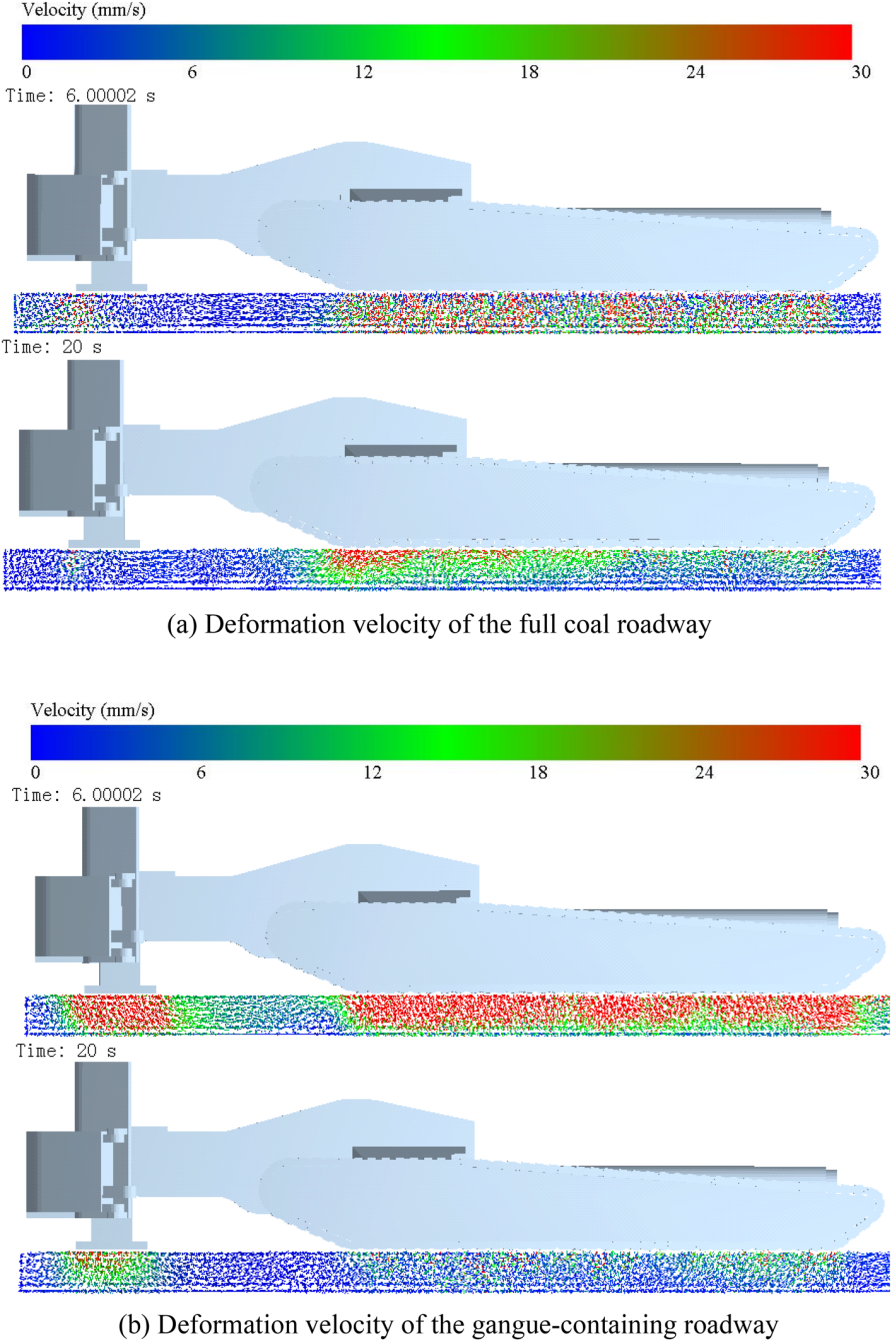
Deformation velocity distribution of the floor.

The pitch attitude of the digging anchor machine during cutting is regulated by two supports at the rear of the machine. The front shovel does not provide support because it can expand and contract on its own during the cutting process to load the fallen coal rock. The deformation of the floor is caused by extrusion and shearing of the upper track and rear support, so to analyse the floor deformation, we first need to analyse the force distribution of the floor. To analyse the effect of the cutting load on the ground pressure of the digging anchor machine, the maximum cutting load and the steady state of the cutting load are taken as research objects, and the force deformation of the floor at 6 s and 20 s is obtained, as shown in Figs. 12 and 13.

By analysing Fig. 12a, we can see that the stress distribution in the floor of the full coal roadway at 6 s is more uniform than that of the gangue-containing roadway. This is because, at this time, the drum only cuts coal rock, the cutting load is smaller, and the maximum cutting resistance in the x direction and y direction is approximately 200 kN, which has less influence on the grounding end to ground load. According to the pressure vector diagram, the compressive stresses in the floor of the full coal roadway are mainly distributed in 3 locations, namely, the frontmost part of the track, the middle part of the track and the rear end of the track. Furthermore, the more concentrated compressive stresses are closer to rear, and the stresses in the floor below the rear support are mainly distributed in the rear half of the contact plane. The stress distribution on the floor at 20 s in the full coal roadway is similar to that at 6 s. This shows that the pressure changes on the floor at the grounding end during digging anchor machine cutting are small and that there is consistency in the pressure distribution.

By analysing Fig. 12b, the floor stress distribution of the digging anchor machine in the gangue roadway at 6 s presents a law of low before and high after. This is expressed in the figure as the closer the grounding end is to the rear, the redder the colour of floor stress, and the higher the degree of concentration. This is because the maximum cutting resistance of the cutting drum in the x and y directions is close to 300 kN. The high cutting resistance causes the front end of the body to lift, and the contact between the front track and floor decreases, resulting in lower pressure. Moreover, the distributed load of body gravity at the rear grounding position rises, the ground pressure on the rear support and rear end of the track rises, and the stress on the floor increases. Figure 12b shows the highest stress concentration in the floor below the rear support and the lowest stress concentration in the front end of the track.

The force causes deformation of the floor. Figure 13 gives the deformation degree of the floor in the cutting state for the two types of roadways, and the red colour indicates the area where the maximum deformation occurs. The deformation on the floor of the full coal roadway mainly occurs below the rear support at 6 s. At this time, the digging anchor machine is affected by the horizontal cutting resistance, the position moves backwards, and the shearing effect of the track on the floor is greater when sliding aggravates the deformation of the floor. Therefore, the maximum deformation area is located at the rear of the rear support, and the maximum deformation area depth can reach 150 mm. The deformation of the floor below the track is relatively small, the maximum deformation occurs only on the surface of the floor, and the deformation decreases from back to front. At 20 s, the deformation mainly occurs below the rearmost track, and the maximum deformation depth is approximately 150 mm, while the closer the track is to the front of floor, the smaller the deformation depth is.

In gangue-containing roadways, the high horizontal cutting resistance during hollowing leads to a large backwards movement of the digging anchor machine. The shearing effect of the track on the floor increases the deformation of the floor in the horizontal direction, while the increased force in the vertical direction increases the compression degree of the floor. The action of the rear support on the floor is divided into longitudinal compression and backwards pushing, so the overall deformation direction of the floor points to the rear of the digging anchor machine and the deep deformation lags the surface layer. The deformation depth of the floor below the rear support can reach 280 mm at 6 s, and the deformation depth of the floor below the track is 250 mm on average, while the deformation depth of the floor below the middle track is slightly less than this value. The deformation of the floor at 20 s is similar to that of the full coal roadway at 6 s.

#### Ground pressure of the digging anchor machine

Roland proposed that the average maximum track pressure *P*_*mm*_, as an indicator used to evaluate the soft floor passing performance of tracked vehicles, is an important parameter for driving the trafficability of digging anchor machines.

The formula for calculating the average maximum grounding pressure is as follows:

13 $$ P_{mm} = \frac{1.258W}{{2mb\sqrt {pd} }} \times 100 $$

where *W* is the mass of the vehicle; *m* is the number of loading wheels on a single track; *b* is the width of the track shoe; *p* is the pitch of the track shoe; and $d$ is the outer diameter of the loading wheel.

**Figure 14:**
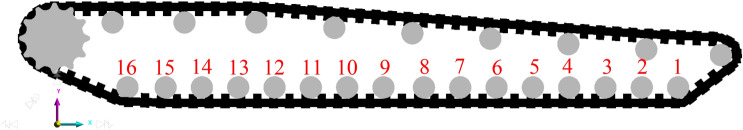
Schematic diagram of the digging anchor machine tracks.

There is a one-to-one correspondence between the peak pressure of the track on the digging anchor machine and the loading wheel under digging conditions^1^, and the pressure value on the loading wheel can be taken as the peak pressure of the track at that place. The digging anchor machine loading wheels are numbered, as shown in Fig. 14.

**Figure 15:**
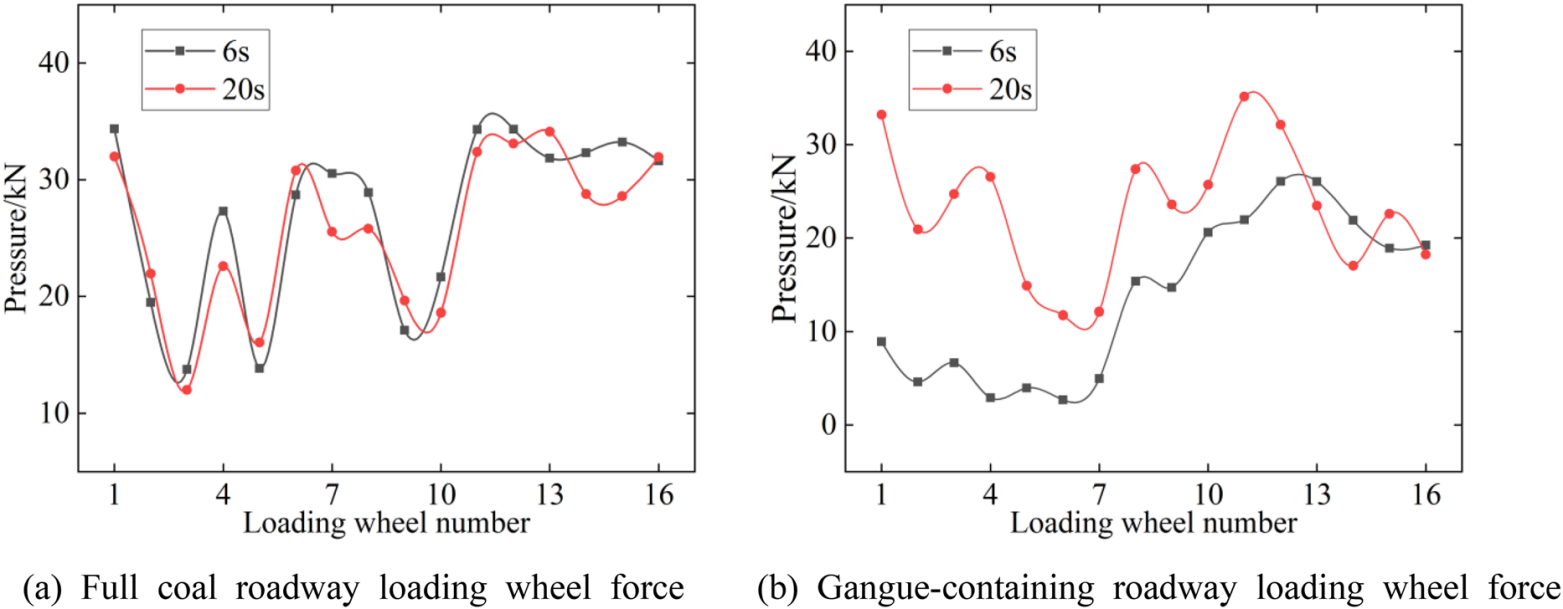
Load bearing wheel force.

Figure 15 and Table 8 give the loading wheel pressure curves at the 6th and 20th roadways, as well as the rear support position pressure, which can be found to be consistent with the stress distribution of the floor. Therefore, it is possible to study the track grounding pressure problem with the help of loading wheel pressure data. According to Fig. 15a, when the digging anchor machine cuts coal rock, the pressure distribution does not change. The layout of the front end, middle part and back half, up to 35 kN and 5–10 kN higher than that of walking, is presented. An increase in the load occurs when cutting gangue; this has a significant effect on the ground pressure distribution of the digging anchor machine. According to Fig. 15b, when gangue exists in the coal wall, the cutting load increases, the ground pressure at the front end of the track decreases to less than 10 kN, and the ground pressure at the rear track decreases to less than 30 kN. The reduced pressure will be transferred to the rear support; at this time, the rear support pressure reaches 200 kN or more, which means the load on the rear support is over 20 t, so the cutting load should be avoided.

**Table 8:** Rear support force.

Time	Full coal roadway	Gangue-containing roadway
6 s	60 kN	200 kN
20 s	38 kN	61 kN

**Figure 16:**
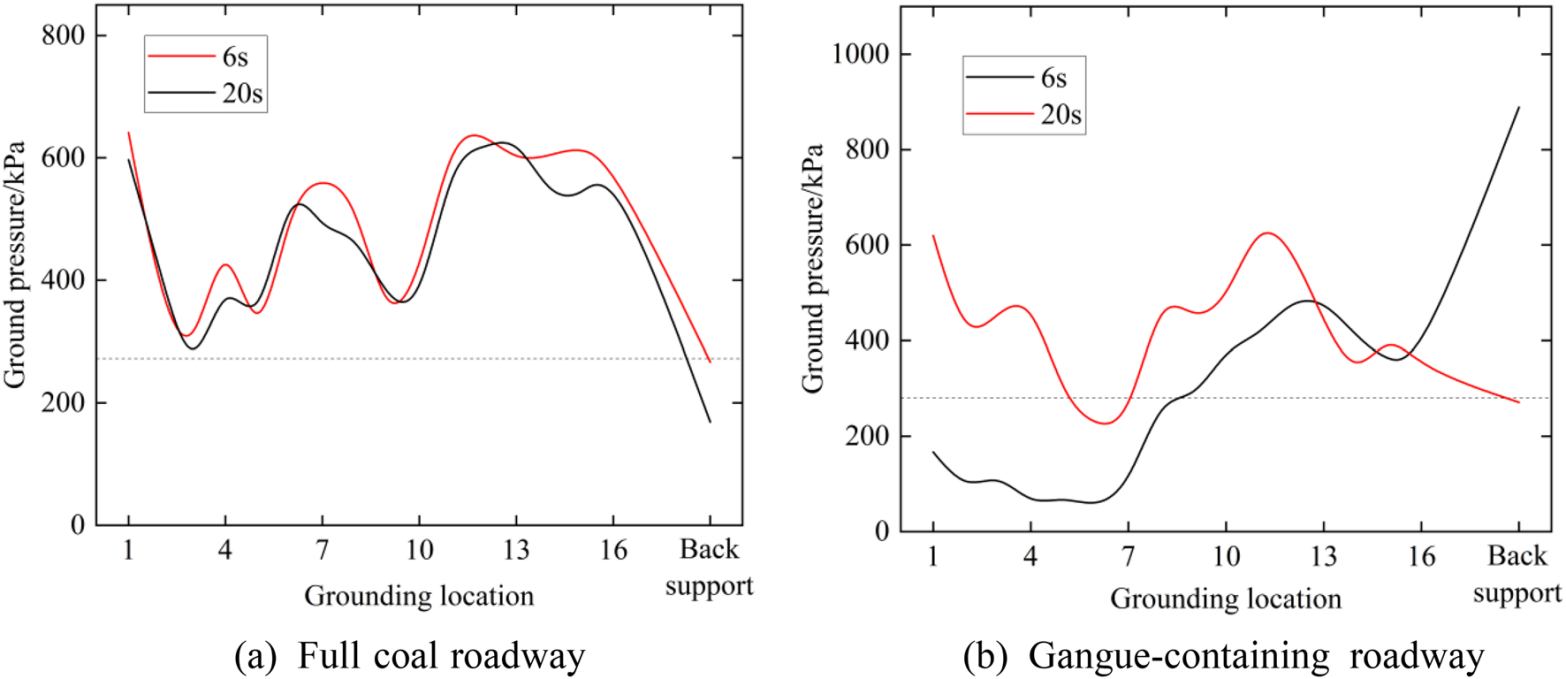
Digging anchor machine ground pressure.

Figure 16 shows the ground pressure of the track and rear support at 6 s and 20 s. The horizontal reference line in the figure is the theoretical ground pressure of the digging anchor machine. This shows that in addition to the 20 s gangue-containing roadway, the ground pressure of the other three working conditions is much higher than the theoretical value, and the highest ground pressure can reach 2.2 times the theoretical value, which is 610 kPa, while the ground pressure value of the rear support is under the theoretical value. Different from the above three working conditions, the ground pressure of gangue-containing roadway at 6 s exhibits a wavy distribution, the ground pressure below the No. 1 loading wheel is 180 kPa, the ground pressure below the Nos. 4–6 loading wheels is only 60 kPa, the ground pressure below Nos. 11–14 loading wheels is more than 400 kN, the ground pressure of No. 15 and No. 16 tracks is reduced to below 300 kN, and the ground pressure at the rear support is as high as 880 kPa. The presence of the rear support reduces the pressure of the track on the floor but aggravates the damage to itself and to the floor where it is located.

## Track shoe soil trough simulation test

**Figure 17:**
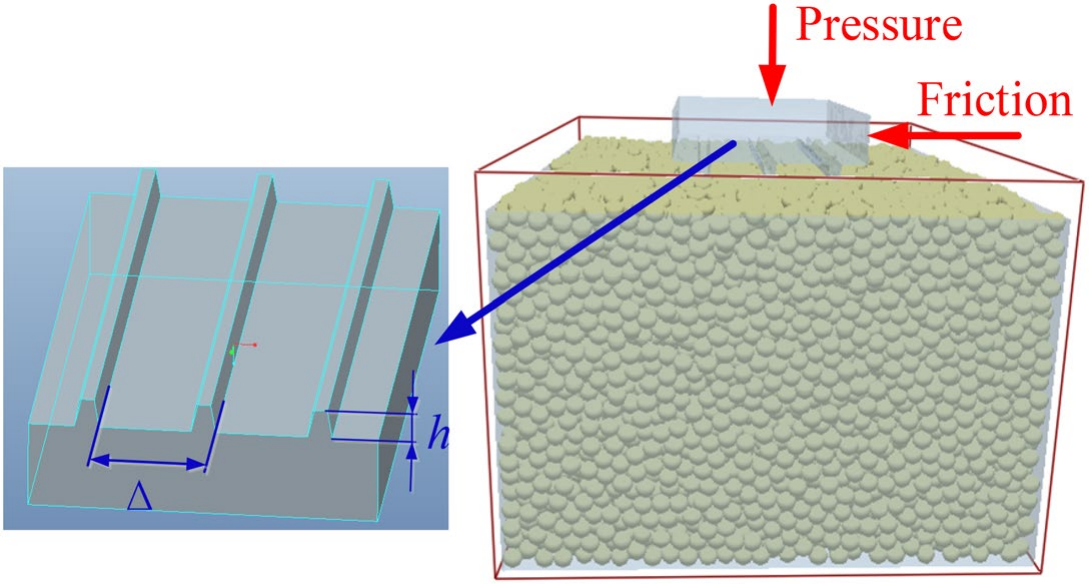
Schematic diagram of the soil trough test.

The effect of the grouser height and grouser spacing on the shear distance is investigated by establishing a shear simulation test of a flat floor in combination with the force on the track shoe during the cutting process. The main body of the track shoe is a rectangular body of 600 mm × 120 mm × 30 mm. To avoid the influence of the number of grousers on the analysis results, Fig. 17 shows that three grousers are set under the main body, and the cross section of the grousers is an isosceles trapezoid with a lower bottom length of 5 mm and a height of *H*_*c*_. The angle between the trapezoid waist and bottom edge is 100°, and the grouser interval is Δ. The variables of the grousers involved in the simulation are shown in Table 9.

**Table 9:** Grouser parameters.

Simulation number	Grouser height h/mm	Grouser interval Δ/mm
1	10	40
2	15	40
3	20	40
4	10	30
5	10	50

### Simulation setup

**Figure 18:**
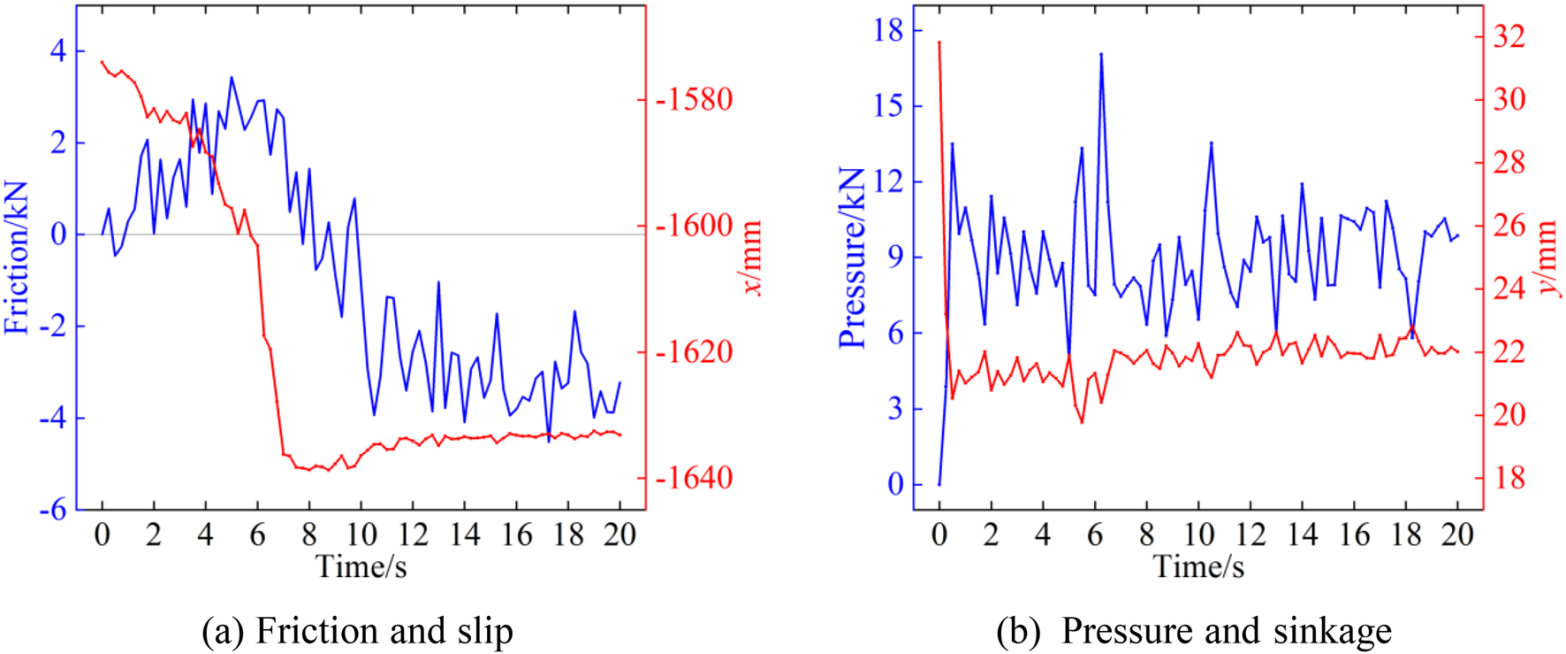
Force versus displacement curve of the No. 12 track shoe.

The floor shear test is an important test method for studying the interaction between soil and machines. To investigate the backwards movement of digging anchor machine tracks during cutting, horizontal and vertical forces are applied to the track shoes to study the degree of shear damage to the floor in the horizontal direction by different parameters of grousers. According to the track ground pressure distribution, the force at the rearmost track shoe at 5–7 s is used as input; at this time, the displacement of the track shoe in the horizontal direction is at a maximum, and the curve of the force and displacement change in the track shoe is shown in Fig. 18.

### Analysis of floor force deformation results

**Figure 19:**
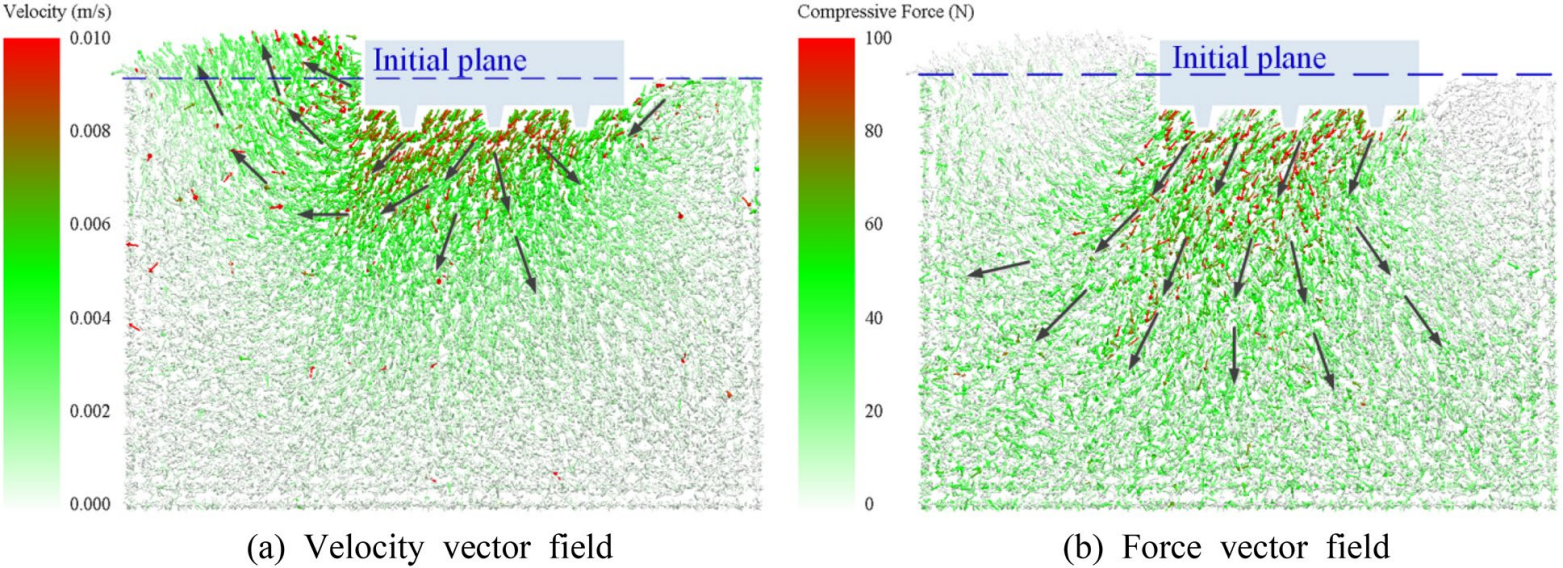
Shear floor velocity and force vector field.

Figure 19 shows the deformation velocity vector of the floor and the force vector inside the floor for a track shoe grouser height of 10 mm and a spacing of 40 mm when the pressure and horizontal force are combined. As seen in Fig. 19a, the track shoe is forced to move downwards and backwards, resulting in the compression of particles below and the accumulation of particles behind. The particle movement is divided into two directions. Part of the particles below the track shoe move downwards with the track shoe, and the speed decreases with increasing depth, while the particles at the rear are pressed to move obliquely downwards and then are blocked by particles at the same depth. This results in the direction of motion changing to oblique upwards and the formation of a pile of particles at the rear of the track. The height of the pile is 20 mm. At the same time, the particles in front of the track shoe are “dragged” by the particles below the track towards the bottom of the track because the floor exhibits particle adhesive properties. The colour of the arrow in the vector diagram represents the movement size. When the track shoe moves backwards, the deformation speed of the floor gradually increases along the backwards direction of the track shoe, and the deformation speed of the frontmost part on the track shoe is the smallest. From Fig. 19b, the pressure in the surface layer is mainly transmitted obliquely downwards, and when the depth reaches 100 mm, the pressure spreads around the surface, and the pressure value gradually decreases from top to bottom. At the same time, the force state of particles near the track shoe is consistent with the velocity field. The force on particles gradually increases along the backwards direction of the track shoe, and the force is mainly concentrated on the side of the grouser near the accumulation area. The pressure on particles at accumulation approaches 0.

### Analysis of the sliding distance

**Figure 20:**
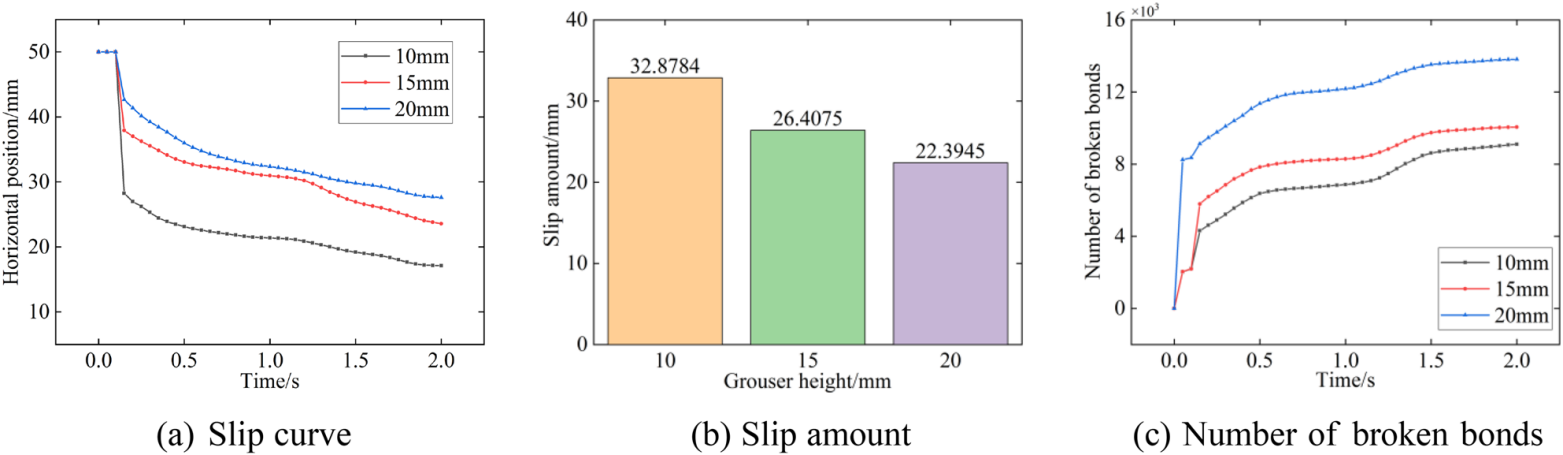
Different grouser heights.

Figure 20 shows the position change curve of the track shoe versus the horizontal position, the final slip amount, and the number of broken bonds for particles. The slip curve with time is approximately a straight line, and the slip distance decreases with increasing grouser height. The maximum sliding distances of the track shoe at the end of 2 s are 32.9 mm, 26.4 mm, and 22.4 mm. The increase in grouser height leads to an increase in track contact area with particles in the horizontal direction, which increases the shear resistance and therefore decreases the track displacement. However, the greater the grouser height is, the greater the damage to the floor during track sliding, and the number of broken bonds increases significantly with increasing grouser height.

**Figure 21:**
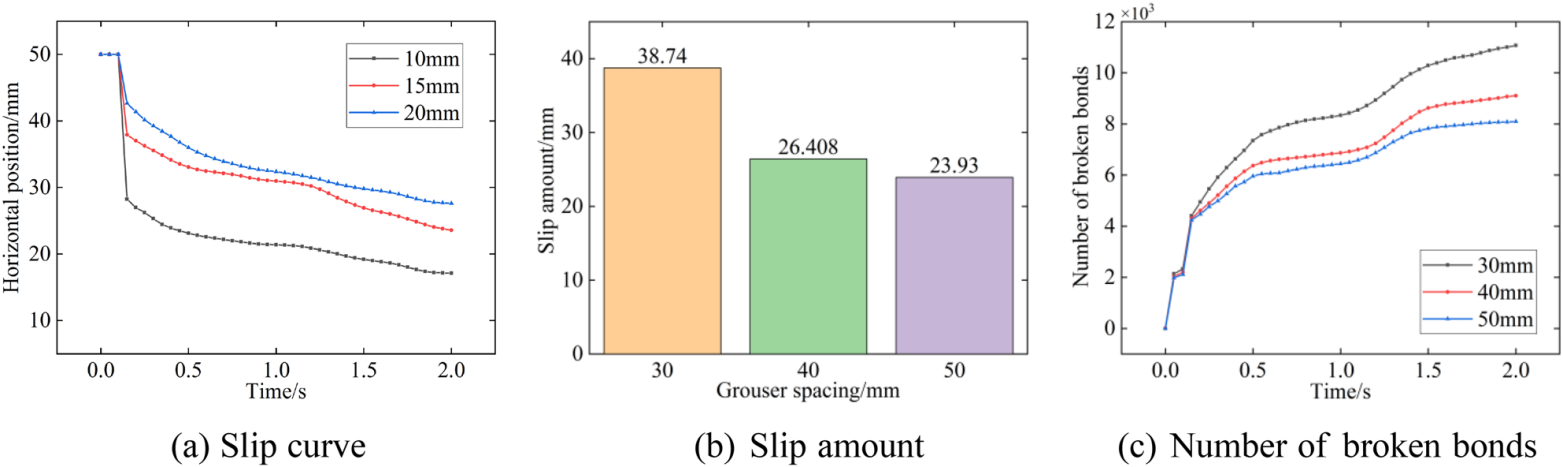
Different grouser spacing.

From Fig. 21, among the three types of grouser spacings, the curve of track horizontal displacement with time is obviously more inclined when the grouser is 30 mm high, which means that the soil trough adhesion is the smallest, the track sliding distance is farther, and the sliding speed is greater. With a grouser spacing of 30 mm, the track shoe slides 38.7 mm at the end of 2 s, which is 1.5 times the spacing of 40 mm and 1.6 times the spacing of 50 mm. This shows that the grouser spacing is too small to provide sufficient traction for the track, and it also increases the damage to the floor. The number of broken bonds in the floor increases as the grouser spacing decreases.

### Analysis of the degree of track shoe wear

**Figure 22:**
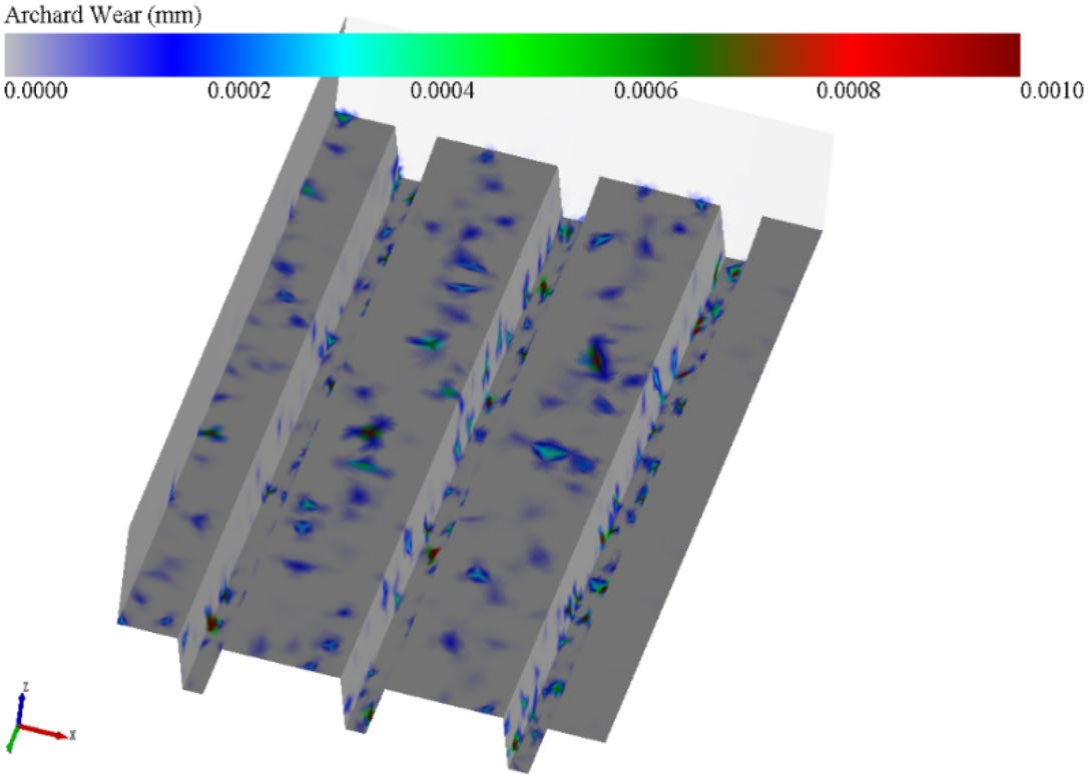
Shear test, track shoe wear cloud.

The track shoe is constantly rubbing against the particles below during the slipping process. Figure 22 shows the wear cloud of the track shoe when the grouser height is 10 mm and the spacing is 40 mm. The left side of the grouser is severely worn, and the right side of the grouser has almost no wear. Combined with Fig. 20, it is found that the particles below this area are under little force and do not act significantly with the track shoe. The left side of the grouser has a higher force and longer contact time with particles than the right side, so this part exhibits relatively more wear, while the top outer edge part of the grouser is the most severely worn and shows the darkest colour.

**Figure 23:**
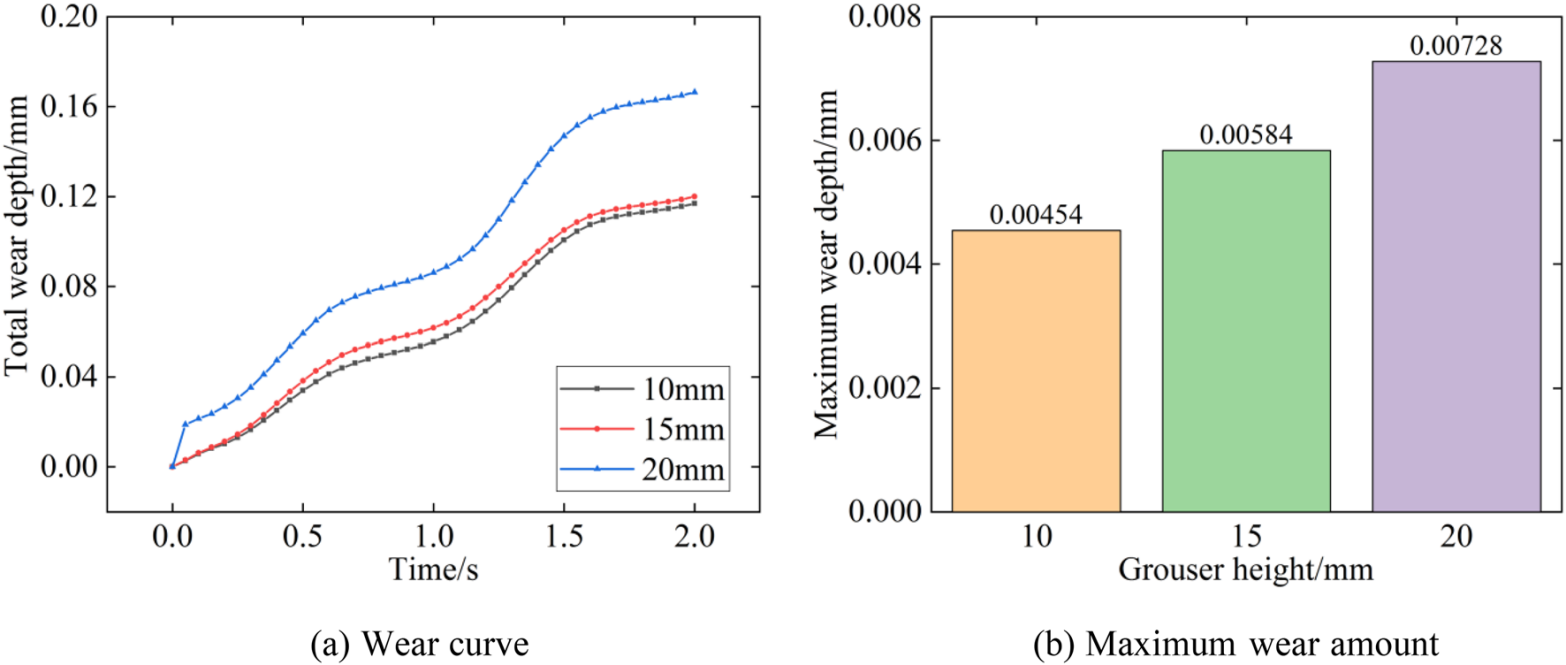
Wear amount with different grouser heights.

Figure 23 shows the relationship between the amount of track shoe wear and the grouser height. As seen from the graph, the total wear amount gradually increases with time, and the curve slope change is influenced by the pressure. When the pressure increases, the resistance of the floor increases, the friction between the particles and track shoe increases, and the wear speed is accelerated. When the grouser height is 10 mm and 15 mm, the total wear depth is 0.12 mm, while when the grouser height increases to 20 mm, the total wear amount is 0.17 mm, which is a 42% increase. The maximum wear depth of the track shoe also increases with the grouser height; the maximum wear depth is 0.0045 mm at 10 mm, 0.0058 mm at 15 mm and 0.0073 mm at 20 mm. As the grouser height increases, the shearing effect of the track on the floor in the horizontal direction increases, and the total and maximum wear amount increases.

**Figure 24:**
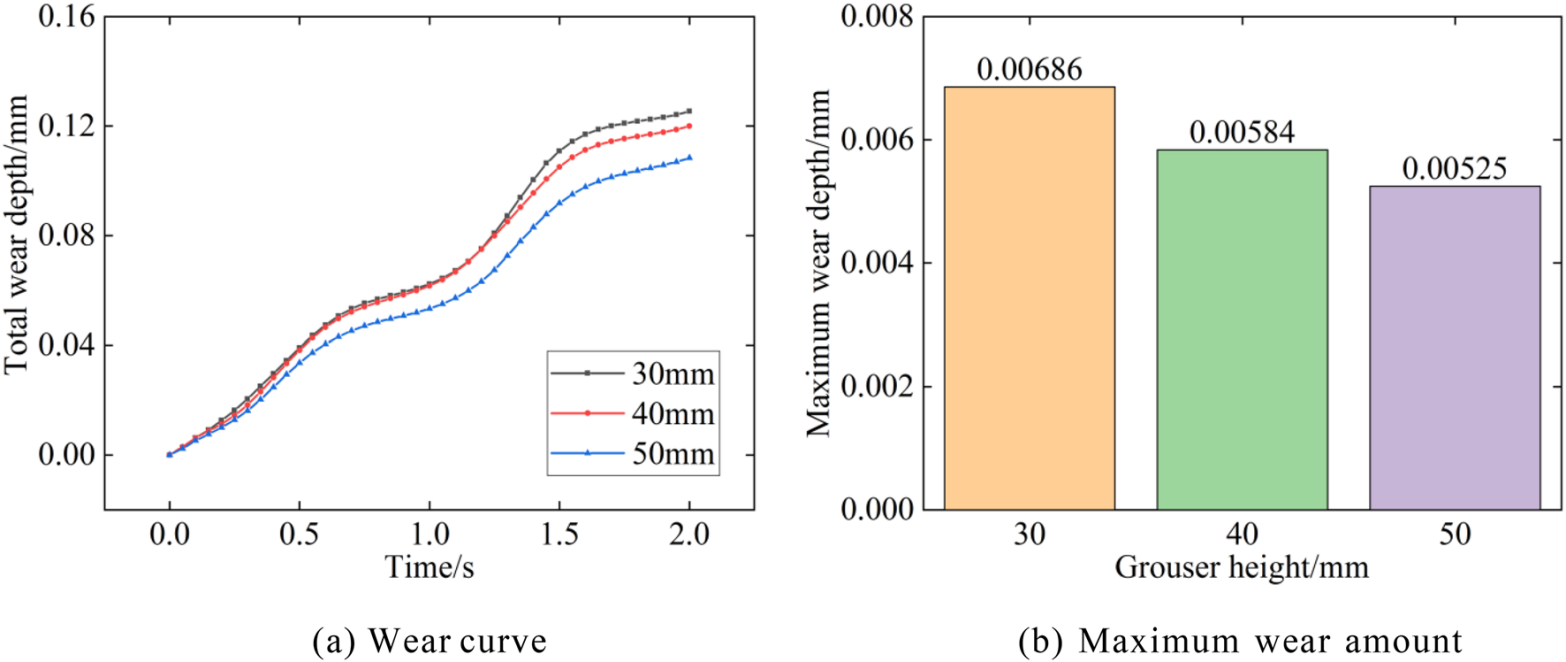
Wear depth with different grouser spacing.

Figure 24 shows the track shoe total wear amount versus the maximum wear amount for different grouser spacings. When the grouser spacing increases, the total and maximum wear amounts of the track decrease. As the grouser spacing increases from 30 to 50 mm, the total amount of wear decreases from 0.13 to 0.12 mm and finally to 0.11 mm, while the maximum track wear depth decreases by 15% and 10%, respectively.

## Conclusion

To reveal the stability of the digging anchor machine and the bearing capacity of the roadway floor under the digging conditions, a Sandvik MB670-1 digging anchor machine is used as a research object in the context of the Zhang Jiamao 5^–2^ coal seam of the Shaanxi Coal and Chemical Industry Group. Based on the two-way coupling theory of DEM-MBD, the dynamics model of the digging anchor machine and the interaction mechanics model between its tracks and the roadway floor are constructed, and the cutting depth of the machine drum, the sliding distance of the track and the stress‒strain of the roadway floor are analysed under the digging conditions. The results of this study can provide a theoretical basis for the control and reliability of the digging anchor machine and the life fatigue prediction of its tracks. The study results are as follows:

When the digging anchor machine drum cuts the full coal wall and the coal wall containing gangue, the simulation shows that the height of the drum centroid during feed hollowing is slightly lower than the theoretical value. However, the depth of drum hollowing varies greatly. The depth of drum hollowing is 484 mm in the full coal roadway and 431 mm in the gangue roadway. Due to the influence of the coal wall containing gangue, the horizontal cutting load of the drum increases, which leads to a reduction of nearly 10% in the hollowing depth of the digging anchor machine drum in the gangue roadway compared to the full coal roadway under digging conditions.

Under two different coal rock cutting conditions, the cutting load generated by the interaction between the drum and coal rock has a negligible effect on the lateral displacement of the digging anchor machine and a significant difference on the displacement in the traction direction. Under the condition of cutting the full coal wall, the digging anchor machine is moved back 10 mm in the traction direction, and under the condition of cutting the coal wall containing gangue, the digging anchor machine is moved back 65 mm. The simultaneous action of the vertical directional load and shear load of the digging anchor machine track leads to the deformation of the roadway floor. Under the condition of cutting the full coal wall, the deformation of the roadway floor basically occurs at the surface of the floor, and the maximum deformation depth is 150 mm. Under the condition of cutting the coal wall containing gangue, due to the increase in drum load, the damage to the roadway floor by digging anchor machine tracks is increased, and the main deformation position of the roadway floor is located at the rear of digging anchor machine. The maximum deformation position is at the rear support of digging anchor machine, and the deformation depth is 280 mm.

Influenced by the shear motion between the digging anchor machine tracks and the roadway floor, the wear of the tracks is aggravated by the combined effect of vertical and shear loads. Meanwhile, to prevent the digging anchor machine backwards distance from being too large under the digging conditions, improve the digging efficiency and increase the friction between the tracks and the floor, the grouser height of the tracks can be increased appropriately. However, as the grouser height increases, the degree of track damage to the floor increases, as does the wear of the tracks.

## Data Availability

The datasets generated and analyzed during the current study are not publicly available due to the research in this paper mainly relies on an important project to carry out the research, which has not yet been completed, so the relevant research data are not yet public. But it can available from the corresponding author on reasonable request.
